# Telemedicine and peritoneal dialysis: the future is now

**DOI:** 10.1590/2175-8239-JBN-2022-E007en

**Published:** 2022-08

**Authors:** Natália Maria da Silva Fernandes, Marcia Regina Gianotti Franco

**Affiliations:** 1 Universidade Federal de Juiz de Fora, Juiz de Fora, MG, Brazil

Resolution no. 2.314/2022^1^ of the Federal Board of Medicine (CFM) regulated and defined telemedicine in Brazil as medical services delivered through the mediation of technology and communication platforms. The current Resolution supersedes Resolution no. 1.643/2022^2^. Face-to-face consultation with a physician remains as the gold standard of care. In Brazil, telemedicine is defined as “the exercise of medicine mediated by digital information and communication technologies for purposes of providing care, education, research, prevention of disease and injury, management and promotion of health”^1^. It can be delivered in real time (synchronous, live sessions) or through asynchronous sessions (offline). Telemedicine may be further divided into seven different modes of care (tele-appointment, tele-consultation, tele-diagnostics, tele-surgery, tele-surveillance, and tele-screening), all of which are subject to inspection by organizations accredited by the Medicine Federal Board based on the ethical principles that govern the physician-patient relationship. During the pandemic, the Brazilian Society of Nephrology designed recommendations covering good practices in dialysis, including telemedicine for peritoneal dialysis^3^.

On the subject of telemedicine and peritoneal dialysis, the term “telemedicine” was only incorporated as a keyword in medical libraries in 1993, and the number of studies comprising the two terms began to grow in 2017 and peaked in 2020, as a consequence of the COVID-19 pandemic. The COVID-19 pandemic showed that telemedicine might be an important element in medical care by providing thousands of patients with access to care. A study by Tabuti et al.^4^, “The impact of telemedicine in the metabolic management and hospitalization of patients on peritoneal dialysis during the COVID-19 pandemic: a national multicenter cohort study”, published in this issue of our journal, investigated the impact of telemedicine on metabolic and hospitalization endpoints in nine clinics with a combined 747 patients. This is an important study, since telemedicine has been definitively incorporated into medical practice and emerged as a subject in the training provided to future physicians attending medical schools. In this retrospective cohort study, the authors divided the patients into three time-related categories, in two months prior to the implementation of telemedicine, one month during the transition into telemedicine, and two months after the implementation of telemedicine. The urgent nature of the implementation of telemedicine during the pandemic led to the development of various modes of delivery based on the availability of clinical resources. The authors looked into classical variables pertaining to the management of patients on peritoneal dialysis.

The first point to consider is the time of observation, which was short for metabolic endpoints and hospitalization, although the latter was negatively impacted. Studies performed in China and Italy, two epicenters of the pandemic, assessed the impact of telemedicine on the outcomes of patients on peritoneal dialysis. In Italy, at the start of the pandemic, in the heavily affected region of Emilia-Romagna, Scarpioni et al.^5^, studied five patients and found that individuals on dialysis were particularly vulnerable and more susceptible to negative outcomes. After 300 videoconferences held during the course of two months, the authors found that the identification of vulnerable patients and telemedicine decreased the incidence of COVID-19 and peritonitis, revealing short-term benefits for telemedicine. In 2020, at the start of the pandemic, Ronco et al.^6^ published recommendations about the use of telemedicine and remote management tools, reporting that optimal care had been administered to patients on peritoneal dialysis without a significant increase in complications or technical failure. The authors supported efforts to prescribe peritoneal dialysis targeting high quality of care. In a recent study, Wang (2022)^7^, with similar purposes as Tabuti et al., published different results showing a decrease in the rate of peritonitis and hospitalization of 1,161 patients over a period of two years. The difference might stem from the longer follow-up time of the Chinese study. Since the start of the pandemic, significant efforts have been made in China to develop a platform (Peritoneal Dialysis Telemedicine-assisted Platform Cohort)^8^ designed to provide a better framework for telemedicine in peritoneal dialysis.

The location of clinics is also a relevant factor. Since Brazil is a country of continental proportions, a multicenter study might present different results depending on the geographical location of the care centers. This was not a relevant finding in this study, which interestingly incorporated the characteristics associated with the care centers (except for geographic location) only to find they did not affect the outcomes. Nevertheless, geographic location is an important element, since the pandemic has taken different shades and forms in the country, which have required the adoption of region-specific public health measures.

An interesting observation is the negative effect of the presence of a caregiver. This finding was not discussed, despite sounding paradoxical at first. It is worth mentioning that, in the final model, only diabetes mellitus affected the outcomes, as also seen in a study performed in a different context with patients on peritoneal dialysis^9^.

Finally, the message left by the study (“… the implementation of telemedicine without proper training and equipment, although necessary in the current scenario, might be harmful...”) is extremely relevant. Considering that telemedicine is a reality today, its incorporation in healthcare training must be discussed in the currículo of the various areas involved in patient care to consider time periods beyond the pandemic.

## References

[1] Resolução CFM nº 2.314, de 20 de abril de 2022 (BR) (2022). Dispõe sobre a definição e regulamentação da telemedicina, como forma de
serviços médicos mediados por tecnologias de comunicação.

[2] Resolução CFM nº 1.643, de 07 de agosto de 2002 (BR) (2002). Define e disciplina a prestação de serviços através da
Telemedicina.

[3] Calice-Silva V, Cabral AS, Bucharles S, Moura-Neto JA, Figueiredo AE, Franco RP (2020). Good practices recommendations from the Brazilian Society of
Nephrology to Peritoneal Dialysis Services related to the new coronavirus
(Covid-19) epidemic. J Bras Nefrol.

[4] Talbuti NIM, Pellizari C, Carrascossi H, Cálice-Silva V, Figueiredo A, Gordon GM (2022). Impact of telemedicine on metabolic control and hospitalization
of peritoneal dialysis patients during the COVID-19 pandemic: a national
multicentric cohort study. Braz. J. Nephrol (J Brasil Nefrol).

[5] Scarpioni R, Manini A, Chiappini P. (2020). Remote patient monitoring in peritoneal dialysis helps reduce
risk of hospitalization during Covid-19 pandemic. J Nephrol.

[6] Ronco C, Manani SM, Giuliani A, Tantillo I, Reis T, Brown EA. (2020). Remote patient management of peritoneal dialysis during COVID-19
pandemic. Peri Dial Int.

[7] Wang Z, Yan W, Lu Y, Song K, Shen H, Wang Y (2022). Effect of combining conventional and telehealth methods on
managing peritoneal dialysis patients: a retrospective single-center
study. Int J Clin Pract.

[8] Ma T, Yang Z, Li S, Pei H, Zhao J, Li Y, PDTAP Working Group (2022). The Peritoneal Dialysis Telemedicine-assisted Platform Cohort
(PDTAP) study: design and methods. Perit Dial Int.

[9] Klinger M, Madziarska K. (2019). Mortality predictor pattern in hemodialysis and peritoneal
dialysis in diabetic patients. Adv Clin Exp Med.

